# Tracking an Underwater Object with Unknown Sensor Noise Covariance Using Orthogonal Polynomial Filters

**DOI:** 10.3390/s22134970

**Published:** 2022-06-30

**Authors:** Kundan Kumar, Shovan Bhaumik, Sanjeev Arulampalam

**Affiliations:** 1 Department of Electrical Engineering, Indian Institute of Technology Patna, Patna 801103, India; kundan.pee16@iitp.ac.in (K.K.); shovan.bhaumik@iitp.ac.in (S.B.); 2 Maritime Division, Defence Science and Technology (DST) Group, Edinburgh, SA 5111, Australia; 3 Faculty of Engineering, Computer & Mathematical Sciences, The University of Adelaide, Adelaide, SA 5005, Australia

**Keywords:** target motion analysis, bearings-only measurement, Doppler-shifted frequency, unknown measurement noise covariance, orthogonal polynomial, sigma point filters

## Abstract

In this manuscript, an underwater target tracking problem with passive sensors is considered. The measurements used to track the target trajectories are (i) only bearing angles, and (ii) Doppler-shifted frequencies and bearing angles. Measurement noise is assumed to follow a zero mean Gaussian probability density function with unknown noise covariance. A method is developed which can estimate the position and velocity of the target along with the unknown measurement noise covariance at each time step. The proposed estimator linearises the nonlinear measurement using an orthogonal polynomial of first order, and the coefficients of the polynomial are evaluated using numerical integration. The unknown sensor noise covariance is estimated online from residual measurements. Compared to available adaptive sigma point filters, it is free from the Cholesky decomposition error. The developed method is applied to two underwater tracking scenarios which consider a nearly constant velocity target. The filter’s efficacy is evaluated using (i) root mean square error (RMSE), (ii) percentage of track loss, (iii) normalised (state) estimation error squared (NEES), (iv) bias norm, and (v) floating point operations (flops) count. From the simulation results, it is observed that the proposed method tracks the target in both scenarios, even for the unknown and time-varying measurement noise covariance case. Furthermore, the tracking accuracy increases with the incorporation of Doppler frequency measurements. The performance of the proposed method is comparable to the adaptive deterministic support point filters, with the advantage of a considerably reduced flops requirement.

## 1. Introduction

Target tracking of an underwater object in a passive mode is a challenging problem owing to the nonlinear and low information content of the passive measurements, coupled with the complexities of the ocean environment [1,2,3,4,5,6]. A typical passive measurement used in such problems is bearings, and the resulting tracking process is termed bearings-only tracking (BOT), where the objective is to estimate the position and velocity of a moving target using bearings-only measurements. Another type of underwater tracking utilises both bearings and Doppler measurements received from the target and is known as Doppler-bearing tracking (DBT). Due to their importance in underwater target tracking, both BOT and DBT have received considerable attention in the literature [7,8,9,10,11]. Since the main aim in the solution to this problem is to estimate the position and velocity of a moving target, this problem is often known as target motion analysis (TMA) [2,7].

In this paper, we assume that the object is approaching towards the ownship following a nearly constant velocity path. The sonar mounted on the ownship measures only bearings or Doppler and bearings. The TMA for such a scenario is challenging due to the following reasons:

(i) With bearings-only measurements, the system is unobservable until the ownship executes a maneuver.
(ii) In certain TMA applications, fast convergence of the solution is important, which is challenging with passive measurements.
(iii) The measurement equation is highly nonlinear, which limits the ability of the estimator to provide reliable tracking performance.
(iv) Sometimes, the measurement noise covariance is time-varying and unknown.

As we mentioned above, in the passive tracking problem, the measurement equations (both bearings-only and Doppler bearings) are nonlinear, and we need to implement a nonlinear filter to estimate the target’s trajectory. The extended Kalman filter (EKF) [12,13] and its variants were initially applied for such problems [14,15,16,17]. However, they have limitations such as poor estimation accuracy and track divergence, particularly for high initial errors [13,18]. To improve the accuracy of estimation in general, a few deterministic sample point filters such as the unscented Kalman filter (UKF) [19,20], Gauss-Hermite filter (GHF) [21,22], and cubature Kalman filter (CKF) [23,24], etc., were developed. In these filters, the probability density functions are approximated with a few deterministic points and weights, which are propagated and updated using the process and measurement equation, respectively. The Cholesky decomposition operation on error covariance is required to be performed in all such filters. Due to processing software limitations, a round-off error arises, which frequently results in error covariances to be asymmetric and non-positive definite, which ultimately leads to unstable filtering. To circumvent this problem, square root filters [25,26,27] were proposed. Although the square root filters are numerically more stable, they are computationally more expensive.

Very recently, an orthogonal polynomial approximation-based filter was proposed [28], which can be seen as an alternative to the square root filtering. This filter uses an orthogonal polynomial approximation to linearise a nonlinear system, and subsequently the Kalman filtering approach is used. The coefficients of the linearised model are calculated by evaluating an intractable integral using a weighted sum of a few deterministic sample points. When these sample points are generated using the cubature rule, the filter is called cubature orthogonal polynomial-based EKF (CO-EKF), and similarly, when the unscented transform and Gauss-Hermite integration rules are used, the proposed filters are named UO-EKF and GHO-EKF, respectively. These filters are more robust and retain the desirable properties of error covariance matrices during software implementation [28], i.e., they are free from the Cholesky decomposition error. It is reported that the orthogonal polynomial filters provide almost similar accuracy to their square root counterparts with much less computation cost.

In this paper, we reformulate the orthogonal polynomial approximation-based filter proposed in [28] in an alternative way which is more straightforward and easier to follow. Further, we extend the orthogonal polynomial approximation filter in such a way that it can be applied to estimate the states when measurement noise covariance is not known. The proposed filter is also capable of estimating time-varying measurement noise covariance along with the states. We consider two tracking scenarios where the tracking is performed with bearings-only and Doppler-bearing measurements. In the first scenario, the ownship executes an evasive maneuver smoothly, and in the second case, it executes a sharp maneuver. The proposed adaptive orthogonal polynomial filters are implemented and their performance is compared with the corresponding square root filters in terms of (i) root mean square error (RMSE), (ii) percentage track loss, (iii) normalised (state) estimation error squared (NEES), (iv) bias norm, (v) floating point operations (flops) count, and (vi) computation time.

From the simulation results, it is observed that the proposed method tracks the target in both the scenarios even for unknown time-varying measurement noise covariance, and tracking accuracy increases with the incorporation of Doppler frequency measurements. While the percentage of track loss of the developed method is comparable to adaptive deterministic support point filters, the flops requirement of our approach is considerably less. The robustness of the proposed method is tested by assuming a target maneuver with a small and constant turn rate, while the estimators wrongly modelled it as constant velocity. To compensate for this, the estimators use a higher process noise covariance, and it has been shown that the proposed filters work with acceptable accuracy, particularly for DBT.

The remaining part of the paper is organised as follows. An underwater tracking problem is formulated in Section 2. In Section 3, we derive the proposed filter. The simulation results are discussed in Section 4, followed by a brief discussion and conclusions.

## 2. Problem Formulation

### 2.1. System Model

The state vector of a target at time index *k* is given by $X_{k}^{t}={\left[\begin{array}{cccc} X_{1,k}^{t} & X_{2,k}^{t} & {\dot{X}}_{1,k}^{t} & {\dot{X}}_{2,k}^{t} \end{array}\right]}^{{}^{\prime }}$, where ($X_{1,k}^{t},X_{2,k}^{t}$) are the true positions and (${\dot{X}}_{1,k}^{t},{\dot{X}}_{2,k}^{t}$) are the true velocities of the target along the *x* and *y* axis, respectively, and ${}^{\prime }$ represents the transpose of a matrix. Similarly, the state vector of the observer (which is ownship) can be expressed as $X_{k}^{o}={\left[\begin{array}{cccc} X_{1,k}^{o} & X_{2,k}^{o} & {\dot{X}}_{1,k}^{o} & {\dot{X}}_{2,k}^{o} \end{array}\right]}^{{}^{\prime }}$.

The target dynamics in discrete time can be expressed as

(1) $$X_{k}^{t}=FX_{k-1}^{t}+\eta_{k-1},$$

where *F* is the state transition matrix. The process noise, $\eta_{k-1}$, follows a Gaussian distribution with zero mean and covariance,

$$Q_{k-1}=\tilde{q}\left[\begin{array}{cc} \;\frac{T^{3}}{3}I_{2\times 2} & \frac{T^{2}}{2}I_{2\times 2}\\ \frac{T^{2}}{2}I_{2\times 2} & TI_{2\times 2} \end{array}\right],$$

where $\tilde{q}$ is the process noise intensity, and *T* is the sampling time. We define the relative state vector as

(2) $$X_{k}=X_{k}^{t}-X_{k}^{o}.$$

Using Equation (1), we can write the state equation as

(3) $$\begin{array}{cc} X_{k} & =FX_{k-1}^{t}+\eta_{k-1}-X_{k}^{0}\\ & =F\left(X_{k-1}+X_{k-1}^{o}\right)-X_{k}^{0}+\eta_{k-1}\\ & =FX_{k-1}-U_{k-1,k}+\eta_{k-1}, \end{array}$$

where $U_{k-1,k}=X_{k}^{0}-FX_{k-1}^{0}$ is known as the vector of deterministic inputs [7]. Here, we consider a single target which follows (i) a nearly constant velocity path described by a constant velocity (CV) model, or (ii) a maneuvering trajectory described by a constant turn (CT) model with constant and known turn rate. It is interesting to note that, for both cases, the process equation is linear.

#### 2.1.1. Constant Velocity Model

The state transition matrix for the CV model is given by

(4) $$F=\left[\begin{array}{cc} I_{2\times 2} & TI_{2\times 2}\\ 0_{2\times 2} & I_{2\times 2} \end{array}\right],$$

and the vector of deterministic inputs [2,7] becomes

(5) $$U_{k-1,k}=\left[\begin{array}{c} \;X_{1,k}^{0}-X_{1,k-1}^{0}-T{\dot{X}}_{1,k-1}^{0}\\ \;X_{2,k}^{0}-X_{2,k-1}^{0}-T{\dot{X}}_{2,k-1}^{0}\\ \;{\dot{X}}_{1,k}^{0}-{\dot{X}}_{1,k-1}^{0}\\ {\dot{X}}_{2,k}^{0}-{\dot{X}}_{2,k-1}^{0} \end{array}\right].$$

#### 2.1.2. Constant Turn Model

In this case, it is assumed that the target maneuvers with a constant and known turn rate, ω. The state transition matrix of this model is expressed as [23,29,30]

(6) $$F=\left[\begin{array}{cccc} \;1 & 0 & \frac{\sin(\omega T)}{\omega } & -\frac{1-\cos(\omega T)}{\omega }\\ \;0 & 1 & \frac{1-\cos(\omega T)}{\omega } & \frac{\sin(\omega T)}{\omega }\\ \;0 & 0 & \cos(\omega T) & -\sin(\omega T)\\ 0 & 0 & \sin(\omega T) & \cos(\omega T) \end{array}\right],$$

and the expression of $U_{k-1,k}$ becomes

(7) $$U_{k-1,k}=\left[\begin{array}{c} \;X_{1,k}^{0}-X_{1,k-1}^{0}-\frac{\sin(\omega T)}{\omega }{\dot{X}}_{1,k-1}^{0}+\frac{1-\cos(\omega T)}{\omega }{\dot{X}}_{2,k-1}^{0}\\ \;X_{2,k}^{0}-X_{2,k-1}^{0}-\frac{1-\cos(\omega T)}{\omega }{\dot{X}}_{1,k-1}^{0}-\frac{\sin(\omega T)}{\omega }{\dot{X}}_{2,k-1}^{0}\\ \;{\dot{X}}_{1,k}^{0}-\cos\left(\omega T\right){\dot{X}}_{1,k-1}^{0}+\sin\left(\omega T\right){\dot{X}}_{2,k-1}^{0}\\ {\dot{X}}_{2,k}^{0}-\sin\left(\omega T\right){\dot{X}}_{1,k-1}^{0}-\cos\left(\omega T\right){\dot{X}}_{2,k-1}^{0} \end{array}\right].$$

It is to be noted that in the limiting condition ω→0, Equations (6) and (7) reduce to Equations (4) and (5), respectively.

### 2.2. Measurement Model

Measurement sensors are mounted on the ownship and all the sensors are passive so that some specific tactical advantages can be achieved. It is assumed that the sonar mounted on the ownship is only capable of measuring the bearing angle. We assume the following two cases.

#### 2.2.1. Case I—Bearings-Only Measurement

The sensors measure the direction of the target location with respect to true north. Such measurements can be expressed as

(8) $$Y_{k}=\gamma \left(X_{k}\right)+\nu_{\theta_{k}},$$

where $\gamma \left(X_{k}\right)=\tan^{-1}\left(X_{1,k}/X_{2,k}\right)$, and $\nu_{\theta_{k}}$ is noise in bearing measurement, which is assumed to be zero mean, white Gaussian with standard deviation, $\sigma_{\theta }$, i.e., $\nu_{\theta_{k}}∼N\left(0,\sigma_{\theta }^{2}\right)$. For such measurements, the system is unobservable [2,7] and the estimation of the target state is only possible when the ownship starts maneuvering. It is to be noted that even if, sometimes, the sensor measures the bearing angle with respect to its own local coordinates, it is easy to transform the measurement with respect to true north.

#### 2.2.2. Case II—Doppler-Bearing Measurement

As the target is moving, a Doppler shift occurs on the tonal frequency emitted from the target, which can also be measured. In such cases, both the noise-corrupted bearing angles and Doppler-shifted frequencies are available. This is popularly known as Doppler-bearing (DB) measurement and the overall measurement function $\gamma (X_{k})$ becomes

(9) $$\gamma \left(X_{k}\right)=\left[\begin{array}{c} \;\tan^{-1}\left(X_{1,k}/X_{2,k}\right)\\ f_{T}\left(1-\frac{{\dot{X}}_{1,k}X_{1,k}}{cr_{k}}-\frac{{\dot{X}}_{2,k}X_{2,k}}{cr_{k}}\right) \end{array}\right],$$

where $f_{T}$ is the constant tonal frequency emitted from the target, $r_{k}^{2}=X_{1,k}^{2}+X_{2,k}^{2}$, *c* is the speed of the sound in water, and the measurement noise vector in this case is $\nu_{k}={\left[\begin{array}{cc} \nu_{\theta_{k}} & \nu_{f_{k}} \end{array}\right]}^{{}^{\prime }}$. Note that $\nu_{f_{k}}$ is the sensor noise in the Doppler measurement and assumed to be zero mean, white, Gaussian with variance $\sigma_{f}^{2}$, i.e., $\nu_{f_{k}}∼N\left(0,\sigma_{f}^{2}\right)$. Further, we assume that the noises in bearing and Doppler measurements is uncorrelated, with overall measurement noise covariance $R_{k}=\mathrm{diag}\left(\sigma_{\theta }^{2},\sigma_{f}^{2}\right)$.

It is to be noted that for Doppler-bearing (DB) measurements, the system is observable and maneuvering of the ownship is not required for estimators to converge [9]. Further, it is expected that if noise variance for Doppler measurement is reasonably low, the Doppler-bearing tracking (DBT) should provide more accurate results than bearings-only tracking (BOT).

## 3. Tracking Methodology

In this section, we formulate a tracking filter for underwater TMA when sensor noise covariance is unknown. The proposed filter linearises the nonlinear measurement function using a first-order orthogonal polynomial approximation with a Gaussian measure as a weight function and estimates the measurement noise covariance $R_{k}$ at each step.

### 3.1. Orthogonal Polynomial Filter

The proposed orthogonal polynomial filter is a kind of linearised Kalman filter, where, unlike the extended Kalman filter, the linearisation is not done using the Taylor series; rather, it is done with a first-order Hermite polynomial. The method was developed for a general nonlinear state estimation problem in our earlier publication [28]. Here, we provide a more straightforward derivation of the filter for an underwater tracking problem.

The problem considered here consists of a linear process model, so the time update step can be done by the Kalman filter (KF). The available measurements are assumed to be (i) bearings, (ii) bearings with Doppler-shifted frequencies. Thus, our measurement $Y_{k}\in R^{n_{y}}$, where $n_{y}$ is 1 or 2 for bearings and Doppler-bearing measurements, respectively. As the measurement equation is nonlinear, it is linearised with the first-order Hermite polynomial, and subsequently the KF scheme is applied for the measurement update.

Let us assume an arbitrary function of Gaussian random variable (r.v.) X, f(X), which maps X to a real space of arbitrary dimension $n_{p}$, i.e., $f\left(X\right):R^{n_{x}}\to R^{n_{p}}$ is approximated with a first-order Hermite polynomial [28,31,32,33]; so,

(10) $$f\left(X\right)\approx A_{0}H_{0}\left(X\right)+A_{1}H_{1}\left(X\right),$$

where the coefficients $A_{0}$ and $A_{1}$ are matrices with dimension $n_{p}\times 1$ and $n_{p}\times n_{x}$, respectively. $H_{0}\left(X\right)$ and $H_{1}\left(X\right)$ are the zero and first-order Hermite polynomial, respectively. Please note that in Equation (10), we neglect higher-order terms so that the function is approximated by a linear equation. All the terms with higher-order Hermite polynomials that are neglected in Equation (10) combined together form the residual error. We choose the Hermite polynomial since its weighting function expansion is the same as the Gaussian [31,32]. As the Hermite polynomial is orthogonal [33],

(11) $$<H_{i}\left(X\right),H_{j}\left(X\right)>=\left\{\begin{array}{cc} I_{n_{x}} & \mathrm{for}\;i=j,\\ 0 & \mathrm{otherwise}, \end{array}\right.$$

where <,> represents the dot product, $X∼N(\hat{X},P)$, and $I_{n_{x}}$ is the identity matrix of dimension $n_{x}$. The coefficient $A_{i}$ can be calculated as

(12) $$\begin{array}{cc} A_{i} & =<f\left(X\right),H_{i}\left(X\right)>\\ & =\int_{-\infty }^{\infty }f\left(X\right)H_{i}{\left(X\right)}^{\prime }N\left(X,\hat{X},P\right)dX. \end{array}$$

It is easy to verify that $H_{0}\left(X\right)=1$ and $H_{1}\left(X\right)=S^{-1}\left(X-\hat{X}\right)$, where $P=SS^{\prime }$. Substituting the values of $H_{0}\left(X\right)$ and $H_{1}\left(X\right)$ in Equation (10), the approximation of f(X) becomes

(13) $$f\left(X\right)\approx A_{0}+A_{1}S^{-1}\left(X-\hat{X}\right).$$

Following Equation (13), we can linearise the measurement function $\gamma (X_{k})$ as

(14) $$\gamma \left(X_{k}\right)\approx A_{k}+B_{k}S_{k|k-1}^{-1}\left(X_{k}-{\hat{X}}_{k|k-1}\right),$$

where the state $X_{k}\in R^{4}$ follows a Gaussian distribution with mean ${\hat{X}}_{k|k-1}$ and covariance $P_{k|k-1}$, and the coefficients $A_{k}$ and $B_{k}$ are $n_{y}\times 1$ and $n_{y}\times 4$ matrices, respectively. $S_{k|k-1}$ is the square root of the covariance matrix $P_{k|k-1}$ (i.e., $P_{k|k-1}=S_{k|k-1}S_{k|k-1}^{\prime })$, which can be calculated by the Cholesky decomposition. Using Equation (12), the coefficient matrices $A_{k}$ and $B_{k}$ can be expressed as

(15) $$A_{k}=\int_{-\infty }^{\infty }\gamma \left(X_{k}\right)N\left(X_{k};{\hat{X}}_{k|k-1},P_{k|k-1}\right)dX_{k},$$

(16) $$\begin{array}{cc} B_{k}= & \int_{-\infty }^{\infty }\gamma \left(X_{k}\right){\left(S_{k|k-1}^{-1}\left(X_{k}-{\hat{X}}_{k|k-1}\right)\right)}^{\prime }N\left(X_{k};{\hat{X}}_{k|k-1},P_{k|k-1}\right)dX_{k}. \end{array}$$

The above integrals cannot be solved analytically for any arbitrary nonlinear function [34]. Various methods such as the Gauss-Hermite (GH) quadrature rule [21], unscented transform [19,20], cubature quadrature (CQ) rule [23,24], and high-degree cubature quadrature (HDCQ) rule [35,36] are available to solve such integrals. Here, in brief, we discuss the GH quadrature rule.

*Gauss-Hermite quadrature rule:* A single-dimensional Gauss-Hermite (GH) quadrature rule [21,34] is given by

(17) $$\int_{-\infty }^{\infty }\frac{1}{\sqrt{2\pi }}f\left(x\right)\exp\left(-x^{2}\right)dx\approx \sum_{i=1}^{m}f\left(\xi_{i}\right)\omega_{i},$$

where $\xi_{i}$ are the quadrature points with associated weights $\omega_{i}$ for i=1,…,m. The above integral can be extended for a multidimensional variable using the product rule [21]. To calculate these quadrature points, we take a symmetric tridiagonal matrix *J*, with zero diagonal elements and $J_{i,i+1}=\sqrt{i/2},1\leq i\leq \left(m-1\right)$ [21,37]. The quadrature points are located at $\xi_{i}=\sqrt{2}\psi_{i}$, where $\psi_{i}$ is the *i*-th eigenvalue of the matrix *J*. The weight $\omega_{i}={\left|v_{i,1}\right|}^{2}$, where $v_{i,1}$ is the first element of the *i*-th normalised eigenvector of *J*. Thus, a multidimensional integral

(18) $$I=\int_{-\infty }^{\infty }f\left(X\right)N\left(X,0,I_{n_{x}}\right)dX$$

can be approximately evaluated as [38]

(19) $$I=\sum_{i_{1}=1}^{m}\cdots \sum_{i_{n_{x}}=1}^{m}f\left(\xi_{i_{1}},\xi_{i_{2}},\cdots ,\xi_{i_{n_{x}}}\right)\omega_{i_{1}}\omega_{i_{2}}\cdots \omega_{i_{n_{x}}}.$$

The total number of required points in the above method for any $n_{x}$ dimensional system is $m^{n_{x}}$. Here, we see that the number of points in the GH quadrature rule increases exponentially with the system’s dimension. For more about the point and weight generation methods of the UKF, CQKF, HDCQKF, readers are referred to [19,21,24,36].

*Calculation of $A_{k}$ and $B_{k}$:* To calculate $A_{k}$ and $B_{k}$ (Equations (15) and (16)) using the above-discussed GH quadrature rule, we need to transform Equations (15) and (16) into standard Gaussian integrals. Transforming the Gaussian r.v. $X_{k}$ into a standard Gaussian r.v. $x_{k}$ following $X_{k}={\hat{X}}_{k|k-1}+S_{k|k-1}x_{k}$, the integrals (Equations (15) and (16)) become

(20) $$A_{k}=\int_{-\infty }^{\infty }\gamma \left({\hat{X}}_{k|k-1}+S_{k|k-1}x_{k}\right)N\left(x_{k};0,I_{4}\right)dx_{k},$$

(21) $$\begin{array}{c} B_{k}=\int_{-\infty }^{\infty }\gamma \left({\hat{X}}_{k|k-1}+S_{k|k-1}x_{k}\right)x_{k}^{\prime }N\left(x_{k};0,I_{4}\right)dx_{k}. \end{array}$$

Using the points ($\xi_{i}$) and weights $\left(\omega_{i}\right)$, the coefficients $A_{k}$ (Equation (20)) can be expressed as

(22) $$A_{k}=\sum_{i=1}^{m}\gamma \left({\hat{X}}_{k|k-1}+S_{k|k-1}\xi_{i}\right)\omega_{i}=\sum_{i=1}^{m}\gamma \left(\chi_{i,k|k-1}\right)\omega_{i},$$

where $\chi_{i,k|k-1}={\hat{X}}_{k|k-1}+S_{k|k-1}\xi_{i}$. Similarly, the $B_{k}$ is calculated as

(23) $$B_{k}=\sum_{i=1}^{m}\gamma \left(\chi_{i,k|k-1}\right)\xi_{i}^{\prime }\omega_{i}.$$

For bearings-only measurements given in Equation (8), $A_{k}=\sum_{i=1}^{m}\tan^{-1}\left(\chi_{\left\{1,i,k|k-1\right\}}/\chi_{\left\{2,i,k|k-1\right\}}\right)$ and $B_{k}=\sum_{i=1}^{m}\tan^{-1}\left(\chi_{\left\{1,i,k|k-1\right\}}/\chi_{\left\{2,i,k|k-1\right\}}\right)\xi_{i}^{\prime }\omega_{i}$, where $\chi_{1,i}$ represents the first element of the *i*-th transformed quadrature point. For the DB measurements given in Equation (9), the expressions of $A_{k}$ and $B_{k}$ become

$$A_{k}=\sum_{i=1}^{m}{\left[\begin{array}{cc} \tan^{-1}\left(\chi_{\left\{1,i,k|k-1\right\}}/\chi_{\left\{2,i,k|k-1\right\}}\right) & {f_{s}}_{k}^{i} \end{array}\right]}^{{}^{\prime }}\omega_{i},$$

$$B_{k}=\sum_{i=1}^{m}{\left[\begin{array}{cc} \tan^{-1}\left(\chi_{\left\{1,i,k|k-1\right\}}/\chi_{\left\{2,i,k|k-1\right\}}\right) & {f_{s}}_{k}^{\left(i\right)} \end{array}\right]}^{{}^{\prime }}\xi_{i}^{\prime }\omega_{i},$$

where ${f_{s}}_{k}^{i}=f_{T}\left(1-\frac{{\dot{\chi }}_{1,i,k|k-1}\chi_{1,i,k|k-1}}{c\;r_{k}^{i}}-\frac{{\dot{\chi }}_{2,i,k|k-1}\chi_{2,i,k|k-1}}{c\;r_{k}^{i}}\right)$, $r_{k}^{i}=\sqrt{\chi_{1,i,k|k-1}^{2}+\chi_{2,i,k|k-1}^{2}}$.

**Remark** **1.** *Even with the assumption of Gaussian pdf of states, the approximation arises due to (i) neglecting the terms containing second- and higher-order Hermite polynomials; (ii) approximate evaluation of the coefficients $A_{0}$ and $A_{1}$ by the summation method. However, if the nonlinear function is in polynomial form, the exact evaluation of coefficients can be done with the Gaussian integral [17].*

**Remark** **2.** *On neglecting higher-order Hermite polynomial terms in the approximation (Equation (10)), an accumulative error may occur. It can be compensated by the following approaches [12] (pp. 395–402): (i) increasing the process noise artificially; (ii) multiplying the error covariance by a factor slightly greater than unity. In both cases, the Kalman gain increases and more weight is given to the present innovation, thus compensating for the error which occurs during linearisation.*

**Remark** **3.** *From the above discussion, we have seen that $A_{k}$ and $B_{k}$ can be evaluated using the weighted sum of deterministic sample points. If we use the GH quadrature rule [21,34] to obtain the points, the estimation method is called Gauss-Hermite orthogonal polynomial-based EKF (GHO-EKF). The extension EKF is used because the proposed method linearises the nonlinear function (but not with Taylor series expansion). Similarly, when the unscented transform [19] and CQ rule [23,24] are used to generate the points, the filters are called unscented orthogonal polynomial-based EKF (UO-EKF) and cubature orthogonal polynomial-based EKF (CO-EKF), respectively.*

#### Measurement Update

As we have linearised the measurement equation, the measurement update step can be performed with the Kalman filter (KF) algorithm. The estimated value of the measurement and its covariance can be calculated as

(24) $$\begin{array}{cc} {\hat{Y}}_{k|k-1} & =E[\left(\gamma \left(X_{k}\right)+\nu_{k}\right)|Y_{1:k-1}]\\ & =\int_{R^{n_{x}}}\left(A_{k}+B_{k}S_{k|k-1}^{-1}\left(X_{k}-{\hat{X}}_{k|k-1}\right)\right)N\left(X_{k};{\hat{X}}_{k|k-1},P_{k|k-1}\right)dX_{k}\\ & =A_{k}, \end{array}$$

and

(25) $$\begin{array}{cc} P_{k|k-1}^{\mathrm{YY}} & =E[\left(Y_{k}-{\hat{Y}}_{k|k-1}\right){\left(Y_{k}-{\hat{Y}}_{k|k-1}\right)}^{\prime }]\\ & =E[\left(B_{k}S_{k|k-1}^{-1}\left(X_{k}-{\hat{X}}_{k|k-1}\right)+\nu_{k}\right){\left(B_{k}S_{k|k-1}^{-1}\left(X_{k}-{\hat{X}}_{k|k-1}\right)+\nu_{k}\right)}^{\prime }]\\ & =B_{k}S_{k|k-1}^{-1}P_{k|k-1}{\left(S_{k|k-1}^{\prime }\right)}^{-1}B_{k}^{\prime }+R_{k}\\ & =B_{k}B_{k}^{\prime }+R_{k}. \end{array}$$

The cross-covariance between the state and measurement can be calculated as

(26) $$\begin{array}{cc} P_{k|k-1}^{\mathrm{XY}}= & E[\left(X_{k}-{\hat{X}}_{k|k-1}\right){\left(Y_{k}-{\hat{Y}}_{k|k-1}\right)}^{\prime }]\\ & =E[\left(X_{k}-{\hat{X}}_{k|k-1}\right){\left(B_{k}S_{k|k-1}^{-1}\left(X_{k}-{\hat{X}}_{k|k-1}\right)+\nu_{k}\right)}^{\prime }]\\ & =P_{k|k-1}{\left(S_{k|k-1}^{\prime }\right)}^{-1}B_{k}^{\prime }\\ & =S_{k|k-1}B_{k}^{\prime }. \end{array}$$

**Remark** **4.** *Although, throughout our work, we consider the measurement noises to be Gaussian, it may not always be so [30,39,40]. In the case of non-Gaussian noise, it can be approximated by a Gaussian distribution and we can apply the proposed method. However, in this case, since there is a mismatch of noise statistics with the assumed one, robust filters might be helpful. Further, the noise can also be represented as a sum of Gaussians, and Gaussian sum filters [41] with the proposed method may help to achieve the desired results.*

### 3.2. Estimation of Measurement Noise Covariance

The measurement noise considered here is time-varying, zero mean white Gaussian with unknown covariance. In the literature, filtering for such problems is known as adaptive filtering [42,43,44,45,46,47,48]. Several adaptive filters have been proposed based on the KF [42,44], EKF [48], UKF [46,49], CKF [50], and GHF [46]. A few works on the TMA using BOT for unknown time-varying measurement noise are found in [51,52]. In this paper, we have developed a noise adaptation technique using the orthogonal polynomial filter for both BOT and DBT. Here, we derive an expression for online $R_{k}$ estimation compatible with the orthogonal polynomial filter and presented in Lemma 1.

**Lemma** **1.** *The covariance of measurement noise, $R_{k}$ can be expressed as*

(27) $$R_{k}=P_{k|k}^{\mathrm{YY}}+b_{k}P_{k|k}b_{k}^{\prime },$$

*where $P_{k|k}^{\mathrm{YY}}$ is the covariance of residual measurement, $P_{k|k}$ is the posterior error covariance, and $b_{k}=B_{k}S_{k|k-1}^{-1}$*

**Proof.**  The measurement equation using Equation (14) can be written as

(28) $$Y_{k}=A_{k}+B_{k}S_{k|k-1}^{-1}\left(X_{k}-{\hat{X}}_{k|k-1}\right)+\nu_{k}.$$

The expression for the posterior estimate of measurement is

(29) $${\hat{Y}}_{k|k}=A_{k}+B_{k}S_{k|k-1}^{-1}\left({\hat{X}}_{k|k}-{\hat{X}}_{k|k-1}\right).$$

Subtracting Equation (29) from Equation (28), we obtain the residual of measurement

$$Y_{k}-{\hat{Y}}_{k|k}=b_{k}\left(X_{k}-{\hat{X}}_{k|k}\right)+\nu_{k}=b_{k}\epsilon_{k|k}+\nu_{k},$$

where $\epsilon_{k|k}=X_{k}-{\hat{X}}_{k|k}$. The covariance of residual measurement becomes

(30) $$\begin{array}{cc} P_{k|k}^{\mathrm{YY}} & =E[\left(Y_{k}-{\hat{Y}}_{k|k}\right){\left(Y_{k}-{\hat{Y}}_{k|k}\right)}^{\prime }]\\ & =E[\left(b_{k}\epsilon_{k|k}+\nu_{k}\right){\left(b_{k}\epsilon_{k|k}+\nu_{k}\right)}^{\prime }]\\ & =b_{k}P_{k|k}b_{k}^{\prime }+E\left[b_{k}\epsilon_{k|k}\nu_{k}^{\prime }\right]+E\left[\nu_{k}\epsilon_{k|k}^{\prime }b_{k}^{\prime }\right]+R_{k}. \end{array}$$

As $X_{k}$, ${\hat{X}}_{k|k-1}$, and $\epsilon_{k|k-1}$ are uncorrelated with measurement noise $\nu_{k}$, $E\left[X_{k}\nu_{k}^{\prime }\right]=E\left[{\hat{X}}_{k|k-1}\nu_{k}^{\prime }\right]=E\left[\epsilon_{k|k-1}\nu_{k}^{\prime }\right]=0$. Using these relations, we can write the following

(31) $$\begin{array}{cc} E[b_{k}\epsilon_{k|k}\nu_{k}^{\prime }] & =E[b_{k}\left(X_{k}-{\hat{X}}_{k|k}\right)\nu_{k}^{\prime }]\\ & =b_{k}E\left[X_{k}\nu_{k}^{\prime }-{\hat{X}}_{k|k}\nu_{k}^{\prime }\right]\\ & =-b_{k}E\left[\left({\hat{X}}_{k|k-1}+K_{k}\left(Y_{k}-{\hat{Y}}_{k|k-1}\right)\right)\nu_{k}^{\prime }\right]\\ & =-b_{k}E\left[\left({\hat{X}}_{k|k-1}+K_{k}b_{k}\epsilon_{k|k-1}+K_{k}\nu_{k}\right)\nu_{k}^{\prime }\right]\\ & =-b_{k}K_{k}R_{k}, \end{array}$$

where the Kalman gain $K_{k}=P_{k|k}^{\mathrm{XY}}{\left(P_{k|k}^{\mathrm{YY}}\right)}^{-1}$. Further, we can evaluate

(32) $$\begin{array}{c} E\left[\nu_{k}\epsilon_{k|k}^{\prime }b_{k}^{\prime }\right]=E{\left[b_{k}\epsilon_{k|k}\nu_{k}^{\prime }\right]}^{\prime }=-R_{k}K_{k}^{\prime }b_{k}^{\prime }. \end{array}$$

Now,

$$\begin{array}{cc} b_{k}P_{k|k}b_{k}^{\prime }-b_{k}K_{k}R_{k} & =b_{k}\left(I-K_{k}b_{k}\right)P_{k|k-1}b_{k}^{\prime }-b_{k}K_{k}R_{k}\\ & =b_{k}P_{k|k-1}b_{k}^{\prime }-b_{k}K_{k}\left(b_{k}P_{k|k-1}b_{k}^{\prime }+R_{k}\right)\\ & =b_{k}P_{k|k-1}b_{k}^{\prime }-b_{k}P_{k|k-1}b_{k}^{\prime }{\left(b_{k}P_{k|k-1}b_{k}^{\prime }+R_{k}\right)}^{-1}\left(b_{k}P_{k|k-1}b_{k}^{\prime }+R_{k}\right)\\ & =0. \end{array}$$

or,

(33) $$b_{k}K_{k}R_{k}=b_{k}P_{k|k}b_{k}^{\prime }.$$

Substituting Equations (31)–(33) in Equation (30), we have Equation (27). □

From the measurement data sequence, $P_{k|k}^{\mathrm{YY}}$ is calculated using the following equation:

(34) $$P_{k|k}^{\mathrm{YY}}=\frac{1}{k-i_{0}+1}\sum_{i=i_{0}}^{k}\left(Y_{i}-{\hat{Y}}_{i|i}\right){\left(Y_{i}-{\hat{Y}}_{i|i}\right)}^{\prime },$$

where $i_{0}$ is the user’s choice to fix a window length of $\left(k-i_{0}+1\right)$. Substituting Equation (34) into Equation (27),

(35) $$R_{k}=\frac{1}{k-i_{0}+1}\sum_{i=i_{0}}^{k}\left(Y_{i}-{\hat{Y}}_{i|i}\right){\left(Y_{i}-{\hat{Y}}_{i|i}\right)}^{\prime }+b_{k}P_{k|k}b_{k}^{\prime }.$$

The detailed algorithm to implement the proposed adaptive orthogonal polynomial-based filter is presented in Algorithm 1.
**Algorithm 1:** Pseudo code for adaptive polynomial filter for target tracking     *Step 1: Initialisation*  1 Initialise the filter with ${\hat{X}}_{0|0}$, $P_{0|0}$, and $R_{0}$.  2 Compute the sample points $\xi_{i}$, and their respective weights $\omega_{i}$.     *Step 2: Time update*  3 Compute the predicted mean and covariance as
$${\hat{X}}_{k|k-1}=F{\hat{X}}_{k-1|k-1}-U_{k-1,k},$$
$$P_{k|k-1}=FP_{k-1|k-1}F^{\prime }+Q_{k-1}.$$     *Step 3: Measurement update*  4 Compute ${\hat{Y}}_{k|k-1}$, $P_{k|k-1}^{\mathrm{YY}}$, and $P_{k|k-1}^{\mathrm{XY}}$ using Equations (24)–(26).  5 Calculate the Kalman gain, $K_{k}=P_{k|k-1}^{\mathrm{XY}}{\left(P_{k|k-1}^{\mathrm{YY}}\right)}^{-1}$.  6 Estimate the posterior state,
${\hat{X}}_{k|k}={\hat{X}}_{k|k-1}+K_{k}\left(Y_{k}-{\hat{Y}}_{k|k-1}\right).$  7 Compute the posterior error covariance,
$$P_{k|k}=P_{k|k-1}-K_{k}P_{k|k-1}^{\mathrm{YY}}K_{k}^{\prime }.$$     *Step 4: Estimate unknown $R_{k}$*  8 Calculate the posterior estimate of the measurement, ${\hat{Y}}_{k|k}$ using Equation (29).  9 Calculate the residual of measurement as $Y_{k}-{\hat{Y}}_{k|k}$. 10 Calculate the covariance of measurement residual ($P_{k|k}^{\mathrm{YY}}$) using Equation (34). 11 Estimate $R_{k}$ using Equation (35).

### 3.3. Computational Advantage

#### 3.3.1. Alternative to the Square Root Filters

In sigma point filters, to calculate the sigma point at each time step, the Cholesky factorisation of the covariance matrix (P), i.e., $P=SS^{\prime }$, is required. The accumulated round-off error associated with the limited word length arithmetics of the processing software [25,26,27] sometimes leads to an asymmetric, non-positive definite covariance matrix, which forces the filter to stop. To circumvent such a problem, the square root sigma point filters such as square root UKF (SRUKF), square root quadrature Kalman filter (SRQKF), square root CKF (SRCKF), etc., were developed [23,25,26,27]. In square root filtering, at each time step, the square root of *P*, i.e., *S*, is propagated directly using QR decomposition. The QR decomposition guarantees positive definite covariance matrices at the expense of increased computational burden.

The orthogonal polynomial filters are free from such problems and less sensitive to the round-off error and preserve the symmetry and positive definiteness property [28]. Furthermore, the computational cost of this filter is less than the square root filters, which we show using the flops count in the next subsection.

#### 3.3.2. Flops Count

We analyze the computational complexity of the filters by counting the number of floating point operations (flops) [26,28,53]. A flop is defined as one basic algebraic operation, such as addition, subtraction, multiplication, and division, between any two floating point numbers [26,53]. The filter algorithm consists of the following matrix operations: (i) addition, (ii) multiplication, (iii) inverse, and (iv) Cholesky decomposition. The addition of two matrices with dimension m×n requires mn flops. The multiplication of an m×n matrix with n×p matrix requires mp(2n−1) number of flops [53]. The inverse and Cholesky operation of any matrix $A\in R^{n\times n}$ require $n^{3}$ and $n^{3}/3+2n^{2}$ flops, respectively [53]. The QR decomposition of any matrix with dimension m×n using the Householder method requires $2mn^{2}-2/3n^{3}$ flops.

We calculate the flops count for all the filters (both ordinary and adaptive) for BOT and DBT measurements, and these are summarised in Table 1. From the table, we see that the filters with DBT measurements require more flops than those with the BOT. The adaptation of the unknown measurement noise covariance brings an extra computational burden on the filtering algorithm. Among all filters, the EKF has the lowest flops count. The orthogonal polynomial filters such as CO-EKF, UO-EKF, and GHO-EKF have lower flops counts than their square root counterparts (SRCKF, SRUKF, and square root GHF (SRGHF)).

## 4. Simulation Results

### 4.1. Tracking Scenarios

**Figure 1:**
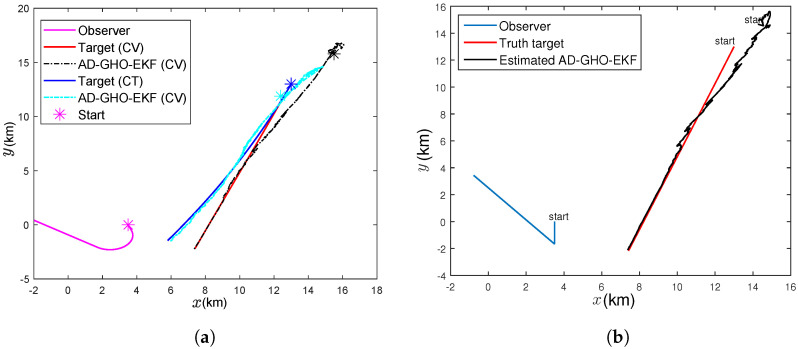
The tracking scenarios: (**a**) moderately nonlinear; (**b**) highly nonlinear.

In this paper, we consider two tracking scenarios, as shown in Figure 1. From the figure, we see that in both scenarios, the target follows a nearly constant velocity path. In the first engagement scenario (Figure 1a), the ownship maneuvers smoothly during the period 61–420 s with a turn rate of 0.4°/s. In the second scenario (Figure 1b), the observer sharply maneuvers at t=181 s and changes its course to 320° from 180°. In the second scenario, the rate of change for the bearing angle during the maneuver is high, which means that it is more difficult for any suboptimal filter to track. It is well known that the maneuvering of ownship makes the system observable. We took two scenarios from the earlier literature [2,7] as benchmark problems so that we could demonstrate the efficacy of our proposed algorithm against the background of existing available results. For both scenarios, the estimators use (i) bearings-only and (ii) bearings and Doppler-shifted frequency measurements, and tracking is performed for 15 min. The speed of sound in water (*c*) is taken to be 1.5 km/s, and the tonal frequency ($f_{T}$) of the target is 385 Hz. Although, in an underwater scenario, the tonal frequency may not be available continuously and it may change after some time interval, here, we assume that the tonal frequency is fixed and available throughout the simulation. The parameters used in the simulation are provided in Table 2.

### 4.2. Performance Metrics

We evaluate the performance of the tracking filters using the following performance metrics.

#### 4.2.1. Root Mean Square Error

The position root mean square error (RMSE) at any time index *k* can be expressed as

$${\mathrm{RMSE}}_{\mathrm{pos},k}=\sqrt{\frac{1}{M}\sum_{i=1}^{M}{\left(X_{1,k}^{i,t}-{\hat{X}}_{1,k}^{i,t}\right)}^{2}+{\left(X_{2,k}^{i,t}-{\hat{X}}_{2,k}^{i,t}\right)}^{2}},$$

where *M* is the number of ensembles, and $\left(X_{1,k}^{i,t},X_{2,k}^{i,t}\right)$ is the position at the *k*-th time step of *i*-th ensemble with corresponding estimated value $\left({\hat{X}}_{1,k}^{i,t},{\hat{X}}_{2,k}^{i,t}\right)$.

#### 4.2.2. Normalised (State) Estimation Error Squared

The normalised (state) estimation error squared (NEES) at the *k*-th time index can be defined as [12] (p. 234)

(36) $${\mathrm{NEES}}_{k}={\left(X_{k}-{\hat{X}}_{k|k}\right)}^{\prime }P_{k|k}^{-1}\left(X_{k}-{\hat{X}}_{k|k}\right).$$

If we assume that the estimators are consistent, and the system is linear with Gaussian noise, then the NEES follows a chi-square distribution with $n_{x}$ degree of freedom, and $E\left[{\mathrm{NEES}}_{k}\right]=n_{x}$, the dimension of the state. The average value of the NEES (ANEES) for *M* Monte Carlo (MC) runs can be calculated as [12] (p. 234) [54]

(37) $${\mathrm{ANEES}}_{k}=\frac{1}{n_{x}M}\sum_{i=1}^{M}{\mathrm{NEES}}_{k}^{i}.$$

The estimators are called consistent if the ${\mathrm{ANEES}}_{k}\in \left[l_{b},u_{b}\right]$, where $l_{b}$ and $u_{b}$ are the lower and upper bounds of the acceptance interval, respectively. Furthermore, the estimator is considered optimistic [12] (p. 245) if ${\mathrm{ANEES}}_{k}>u_{b}$, because, in this case, the value of $P_{k|k}$ is too small, whereas the estimator is pessimistic [12] (p. 245) when the value of ${\mathrm{ANEES}}_{k}$ is lower than $l_{b}$. The calculation of $l_{b}$ and $u_{b}$ can be found in [28,55] and references therein.

#### 4.2.3. Bias Norm

The position bias norm at the *k*-th time step for *M* number of MC runs is expressed as [56]

(38) $$\mathrm{Bias}\;{\mathrm{norm}}_{k}={∥\frac{1}{M}\sum_{i=1}^{M}{\hat{X}}_{p,k}^{i}-\frac{1}{M}\sum_{i=1}^{M}X_{p,k}^{i}∥}_{2},$$

where ${∥\cdot ∥}_{2}$ denotes the vector norm, $X_{p,k}=\left(X_{1,k},\;X_{2,k}\right)$ is the true position, and ${\hat{X}}_{p,k}=\left({\hat{X}}_{1,k},\;{\hat{X}}_{2,k}\right)$ is its posterior estimate.

#### 4.2.4. Track Loss

The terminal error of estimation is defined as

$$e_{b}=\sqrt{{\left(X_{1,kmax}^{t}-{\hat{X}}_{1,kmax}^{t}\right)}^{2}+{\left(X_{2,kmax}^{t}-{\hat{X}}_{2,kmax}^{t}\right)}^{2}},$$

where $X_{1,kmax}^{t}$ and ${\hat{X}}_{1,kmax}^{t}$ are the true and estimated x-position at the final time step, with similar definitions for these variables with subscript 2, which correspond to the y-position. A track is deemed lost if the terminal error ($e_{b}$) is above a threshold, $e_{T}$.

### 4.3. Initialisation of the Filters

For a fair comparison, all the filters are initialised with the same mean and covariance [2] (pp. 115–117), [7]. We initialise the range with a random number $\hat{r}∼N\left(r,\sigma_{r}^{2}\right)$, where *r* is the true initial range and $\sigma_{r}$ is the standard deviation of the range. Similarly, we initialise the first bearing angle to be $\theta_{0}∼N\left(\theta ,\sigma_{\theta }^{2}\right)$ and target speed $\hat{s}∼N\left(s,\sigma_{s}^{2}\right)$, where θ and *s* are the true bearing angle and target speed, respectively. $\sigma_{\theta }$ and $\sigma_{s}$ are the standard deviation of the bearing angle and target speed, respectively. Moreover, in initialising the target course, the filters assume that the target moves towards the observer, so the initial target course estimate is calculated by $\hat{c}=\theta_{0}+\pi$. As our state vector consists of relative positions and velocities, the initial estimate of it can be expressed as ${\hat{X}}_{0|0}={\left[\begin{array}{cccc} \hat{r}\sin\left(\theta_{0}\right) & \hat{r}\cos\left(\theta_{0}\right) & \hat{s}\sin\left(\hat{c}\right)-{\dot{X}}_{1,0}^{0} & \hat{s}\cos\left(\hat{c}\right)-{\dot{X}}_{2,0}^{0} \end{array}\right]}^{\prime },$ where $\left({\dot{X}}_{1,0}^{0},{\dot{X}}_{2,0}^{0}\right)$ is the initial velocity of the observer. The initial error covariance can be written as [2] (pp. 116–117)

(39) $$P_{0|0}=\left[\begin{array}{cccc} P_{\mathrm{XX}} & P_{\mathrm{XY}} & 0 & 0\\ P_{\mathrm{YX}} & P_{\mathrm{YY}} & 0 & 0\\ 0 & 0 & P_{\dot{X}\dot{X}} & P_{\dot{X}\dot{Y}}\\ 0 & 0 & P_{\dot{Y}\dot{X}} & P_{\dot{Y}\dot{Y}} \end{array}\right],$$

where

$$P_{\mathrm{XX}}={\hat{r}}^{2}\sigma_{\theta }^{2}\cos^{2}\left(\theta_{0}\right)+\sigma_{r}^{2}\sin^{2}\left(\theta_{0}\right),$$

$$P_{\mathrm{YY}}={\hat{r}}^{2}\sigma_{\theta }^{2}\sin^{2}\left(\theta_{0}\right)+\sigma_{r}^{2}\cos^{2}\left(\theta_{0}\right),$$

$$P_{\mathrm{XY}}=P_{\mathrm{YX}}=\left(\sigma_{r}^{2}-{\hat{r}}^{2}\sigma_{\theta }^{2}\right)\sin\left(\theta_{0}\right)\cos\left(\theta_{0}\right),$$

$$P_{\dot{X}\dot{X}}={\hat{s}}^{2}\sigma_{c}^{2}\cos^{2}\left(\hat{c}\right)+\sigma_{s}^{2}\sin^{2}\left(\hat{c}\right),$$

$$P_{\dot{Y}\dot{Y}}={\hat{s}}^{2}\sigma_{c}^{2}\sin^{2}\left(\hat{c}\right)+\sigma_{s}^{2}\cos^{2}\left(\hat{c}\right),$$

$$P_{\dot{X}\dot{Y}}=P_{\dot{Y}\dot{X}}=\left(\sigma_{s}^{2}-{\hat{s}}^{2}\sigma_{c}^{2}\right)\sin\left(\hat{c}\right)\cos\left(\hat{c}\right).$$

### 4.4. Performance Comparison

All the filters, namely the orthogonal polynomial filters and deterministic sample point filters, are implemented with BOT and DBT measurements for Scenario I and Scenario II in the following cases: (i) known and time-invariant measurement noise (we call it $R_{\mathrm{known}}$), (ii) known and time-varying measurement noise (we call it $R_{k,\mathrm{known}}$), (iii) unknown and time-invariant measurement noise (we denote it $R_{\mathrm{unknown}}$), (iv) unknown and time-varying measurement noise (we denote it $R_{k,\mathrm{unknown}}$). It is needless to say that we replace the filters with their adaptive counterparts whenever we encounter unknown measurement noise covariance. For the $R_{k,\mathrm{unknown}}$ case, we vary $R_{k}$ from ${\left(1.5{}^{\circ}\right)}^{2}$ to ${\left(4{}^{\circ}\right)}^{2}$ linearly based on the range of target from ownship. Results are summarised in figures and tables showing the RMSE, ANEES, bias norm, track loss, and computational time for various cases. Throughout the simulation, for the SRUKF, we set κ=1 as the choice $\kappa =3-n_{x}$ was found to be problematic due to the negative weights. However, such problems did not appear in UO-EKF and so, for this filter, we set κ=−1. For SRGHF and GHO-EKF, we used three univariate points (i.e., m=3), which resulted in a total of $3^{4}=81$ support points.

#### 4.4.1. Scenario I: Case I—Bearings-Only Tracking

**Figure 2:**
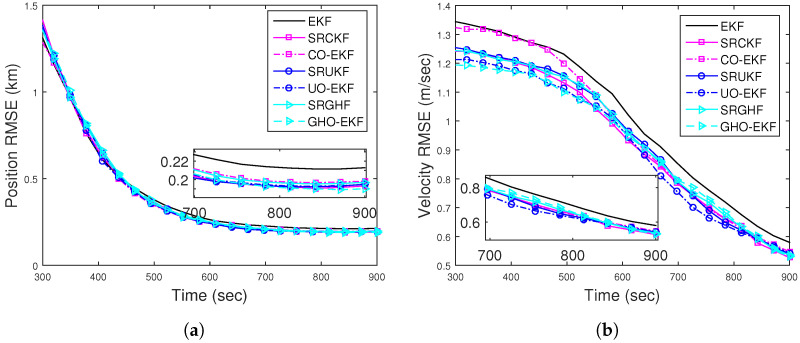
RMSEs of the BOT for Scenario I: (**a**) position; (**b**) velocity.

**Figure 3:**
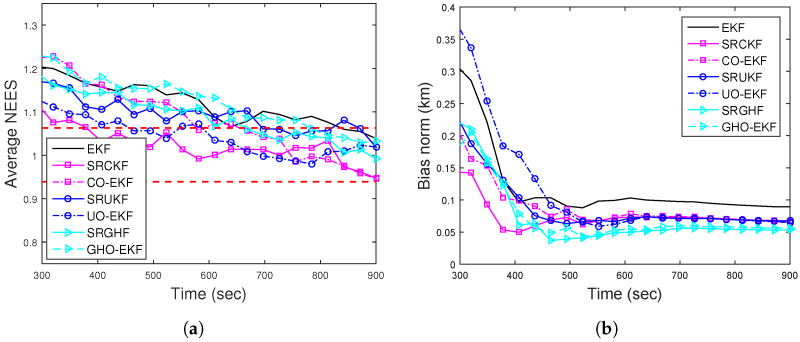
(**a**) ANEES; (**b**) position bias norm of the BOT for Scenario I.

Figure 2a,b show the RMSEs of position and velocity for $R_{\mathrm{known}}$ obtained from 500 MC runs (excluding track loss cases with $e_{T}$ = 500 m). From these figures, we see that except for the EKF, all the filters provide similar RMSE. To see the filters’ consistency, the average NEES (ANEES) obtained from 500 MC runs are plotted in Figure 3a with 95% probability regions, for which the lower bound and upper bound become $l_{b}=0.939$ and $u_{b}=1.0629$, respectively [28,55]. From this figure, we see that all the filters’ ANEES values fall inside the concentration region only by the end of the simulation time. Position bias norms obtained from 500 MC runs are plotted in Figure 3b. From this figure, we see that the bias norms of all filters are sufficiently low, and among them, the SRGHF and GHO-EKF attain the lowest bias norm.

**Figure 4:**
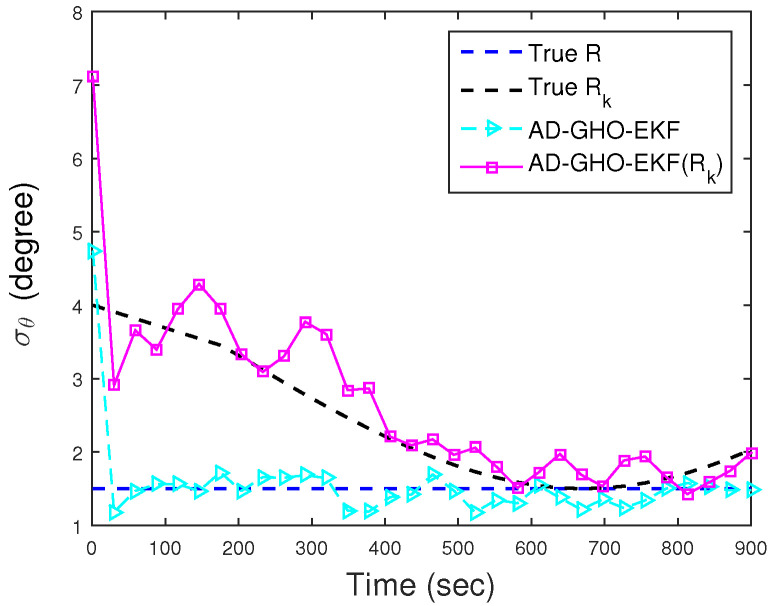
True and estimated *R* (both time-varying and time-invariant) for Scenario I.

**Figure 5:**
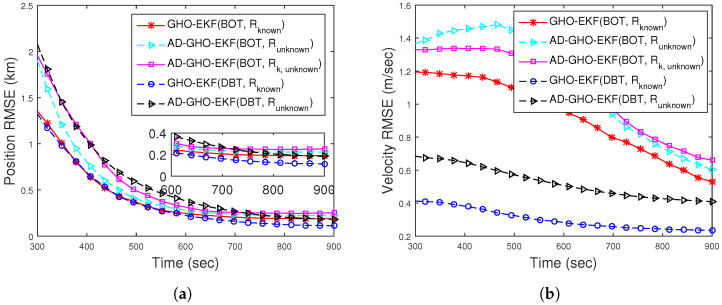
RMSEs of the BOT and DBT for Scenario I for unknown *R*.

To show the efficacy of the proposed measurement noise adaptation technique, in Figure 4, we plot $R_{\mathrm{unknown}}$ and $R_{k,\mathrm{unknown}}$ and their estimates obtained from the adaptive GHO-EKF (AD-GHO-EKF). From this figure, we see that the estimated noise covariances converge to their true values. Very similar estimates are obtained from other adaptive polynomial filters and are not plotted in the figure. To observe the effect of unknown *R* in state estimation (both BOT and DBT cases), position and velocity RMSEs (excluding track loss cases, with error threshold $e_{T}$ = 500 m) obtained from a single representative filter, say AD-GHO-EKF, are plotted in Figure 5a,b, respectively. Here, we also provide the RMSE results for the DBT case in order to show the improvement in RMSE that we could achieve in comparison to BOT if Doppler shifts are incorporated as measurements. The findings of the result for the DBT case are explicitly discussed in Scenario I: Case II. The results are compared with GHO-EKF for both the time-varying and time-invariant unknown measurement noise covariance. From these figures, we see that the GHO-EKF provides lower RMSEs compared to AD-GHO-EKF in this situation. Furthermore, the RMSE for the $R_{\mathrm{unknown}}$ case is slightly less than $R_{k,\mathrm{unknown}}$, as expected.

**Figure 6:**
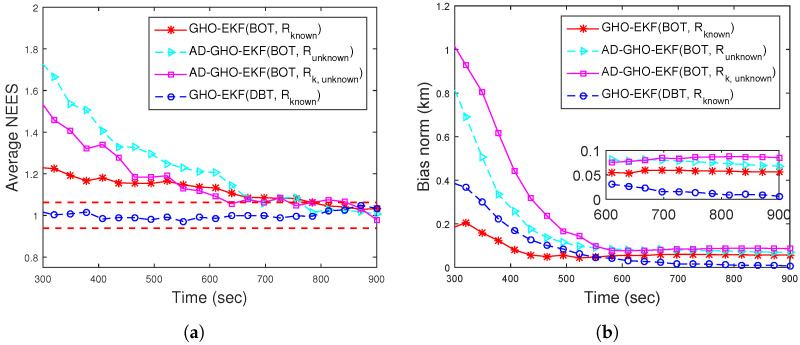
(**a**) ANEES and (**b**) position bias norm for BOT and DBT for Scenario I.

ANEES metrics are calculated for various adaptive filters for $e_{T}\in \left\{200\;\;m,500\;\;m\right\}$. For $e_{T}$ = 500 m, none of the filters’ ANEES is inside the concentration region. In Figure 6a, the ANEES of various adaptive filters are plotted from 500 MC runs $e_{T}$ = 200 m, and for this case, we notice that the ANEES of the GHO-EKF (DBT, $R_{\mathrm{known}}$) is consistently inside the probability region, whereas the same obtained from other filters fall inside the region only after 650 s. The poor ANEES results are mainly due to low observability and unknown time-varying measurement noise covariance. Furthermore, we provide the position bias norm of GHO-EKF and AD-GHO-EKF for unknown measurement noise covariance obtained from 500 MC runs in Figure 6b. From this figure, we observe that GHO-EKF (DBT) has the lowest bias norm, whereas the AD-GHO-EKF for $R_{k,\mathrm{unknown}}$ attains the highest.

**Figure 7:**
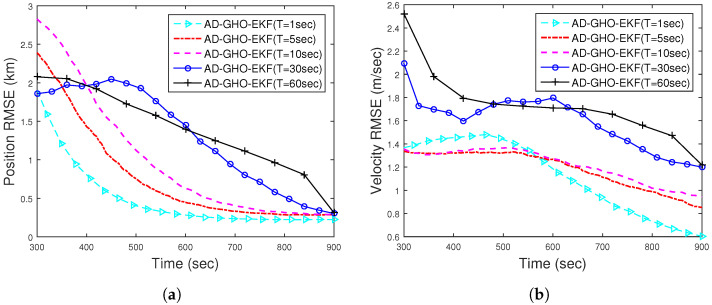
RMSEs of the (**a**) position; (**b**) velocity of the BOT for varying *T* of $R_{\mathrm{unknown}}$ for Scenario I.

Next, we study the effect of sampling time on the accuracy of estimation. In Figure 7, we plot the position and velocity RMSE (excluding the track divergence with $e_{T}$ = 500 m) of AD-GHO-EKF obtained from 500 MC runs for different sampling times, *T*, ranging from 1 s to 60 s. From these figures, we observe that for lower sampling time *T*, the AD-GHO-EKF exhibits better performance, as expected. However, it is to be noted that the sampling time of the sensor cannot be very small as certain time is required to accumulate the energy received in order to measure the bearing angle.

#### 4.4.2. Scenario I: Case II—Doppler-Bearing Tracking

**Figure 8:**
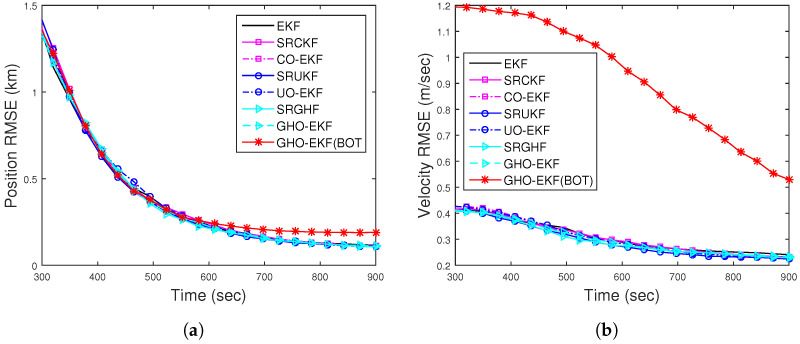
RMSEs of the (**a**) position; (**b**) velocity of the DBT for Scenario I.

**Figure 9:**
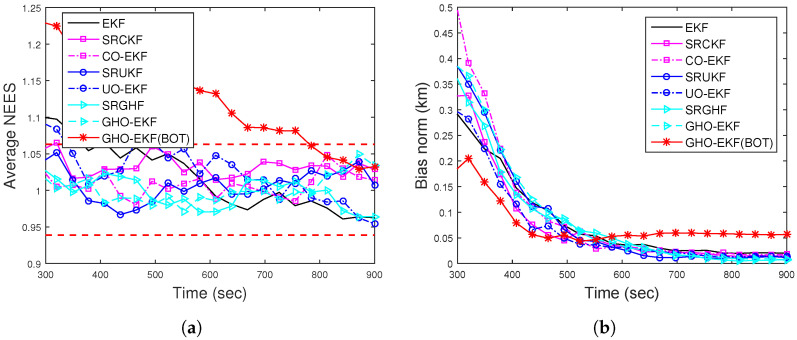
(**a**) ANEES; (**b**) position bias norm of the DBT for Scenario I.

Figure 8a,b show the position and velocity RMSEs for DBT obtained from square root filters and orthogonal polynomial filters out of 500 MC runs. They are calculated with known $R={\left(1.5{}^{\circ}\right)}^{2}$, excluding track divergence cases. All the RMSEs are comparable and lower than the RMSE obtained from BOT. The ANEES with the 500 m track loss threshold are within the provided probability region. The position bias norms of the filters approach zero and are much below that of BOT. From these figures (Figure 8 and Figure 9), we can conclude that the filters with DBT measurements track more effectively than those with BOT measurements.

The efficacy of the proposed adaptive estimator is also checked for $R_{\mathrm{unknown}}$ using DBT measurements. We provide the position and velocity RMSEs of GHO-EKF and AD-GHO-EKF ($R_{\mathrm{unknown}}$) obtained from 500 MC runs (excluding the track divergence cases, $e_{T}$ = 500 m)) in Figure 5. From this figure, we see that GHO-EKF attains lower RMSEs than AD-GHO-EKF. We also see that the incorporation of the Doppler-shifted frequency improves the filters’ estimation accuracy.

Now, we compare the performance of filters (EKF, orthogonal polynomial filters, deterministic square root sample point filters, and their adaptive counterparts) for BOT and DBT in terms of percentage track loss obtained from 10,000 MC runs, presented in Table 3. The track divergence bound is set to $e_{T}=500$ m. From this table, we see that the filters with DBT measurements have lower track loss than those with BOT. As expected, filters with known *R* show better performance than those with unknown *R*, i.e., adaptive filters. The orthogonal polynomial filters provide almost similar track loss as their square root counterparts. We also observe that the filters with time-varying $R_{k}$ (known and unknown) show higher track loss than time-invariant *R*, and these results are not provided. Furthermore, we observe that the track loss count of all the filters increases with the increase in sampling time. This happens due to the fact that with the increase in sampling time, the observability of the system decreases, which further deteriorates the filters’ performance.

#### 4.4.3. Scenario II: Case I—Bearings-Only Tracking

**Figure 10:**
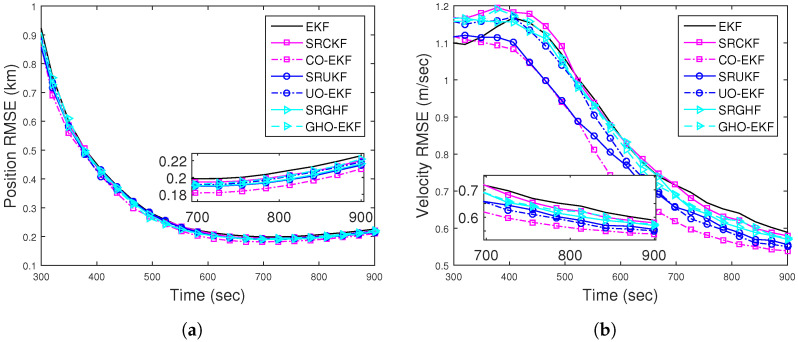
(**a**) Position; (**b**) velocity RMSE of the BOT for Scenario II.

**Figure 11:**
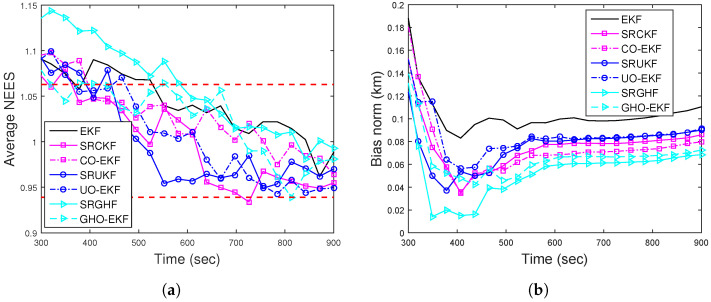
(**a**) ANEES; (**b**) position bias norm of the BOT for Scenario II.

For BOT, the position and velocity RMSE of different filters for $R_{\mathrm{known}}$ from 500 MC runs are plotted in Figure 10a,b, respectively. From these figures, we see that except for the EKF, all other filters attain similar RMSEs. Figure 11a shows that except EKF, the ANEES of all filters (with $e_{T}=500$ m) fall within the provided probability region. The position bias norm plot (Figure 11b) shows that all the filters attain a sufficiently low bias norm.

#### 4.4.4. Scenario II: Case II—Doppler-Bearing Tracking

**Figure 12:**
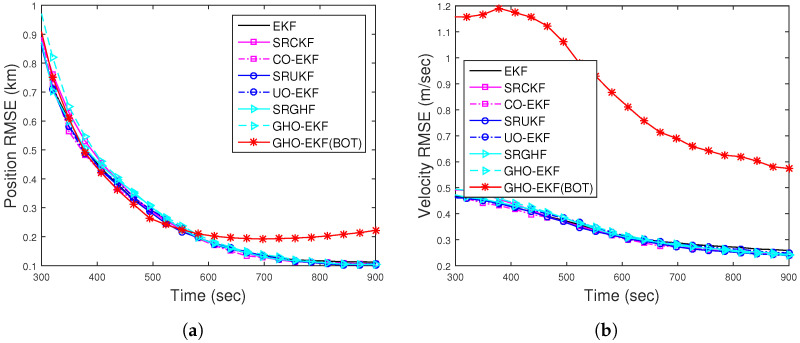
RMSEs of the (**a**) position; (**b**) velocity of the DBT for Scenario II.

For DBT, the position and velocity RMSE of different filters for $R_{\mathrm{known}}$ obtained from 500 MC runs are plotted in Figure 12a,b, respectively. From this figure, we see that the RMSEs of all filters are comparable and lower than the RMSEs obtained from BOT.

**Figure 13:**
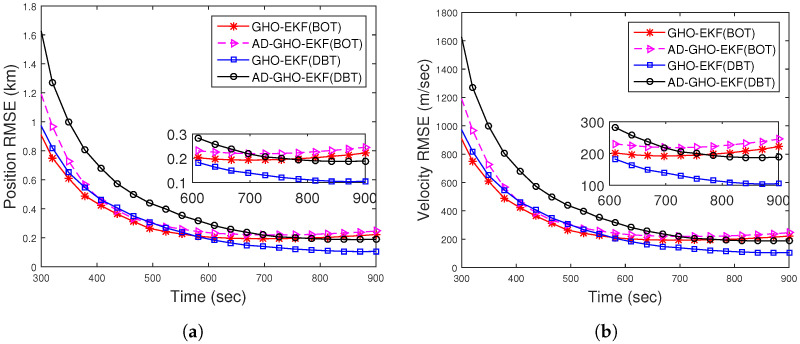
RMSEs of the (**a**) position; (**b**) velocity of the BOT and DBT for Scenario II for unknown fixed *R*.

The estimation accuracy of the proposed AD-GHO-EKF for $R_{\mathrm{unknown}}$ for BOT and DBT is examined in Figure 13 in terms RMSEs (obtained from 500 MC runs). For comparison, we also include the RMSEs of GHO-EKF for $R_{\mathrm{known}}$. From this figure, it can be seen that the GHO-EKF provides better estimation accuracy than the AD-GHO-EKF for both cases of measurements. We also see that the RMSE with DBT measurements is lower than the RMSE obtained from the BOT.

Now, we calculate the percentage track loss of the EKF, square root filters, orthogonal polynomial filters, and their adaptive counterparts out of 10,000 MC runs for BOT and DBT, the results of which are presented in Table 3. The track loss bound is set to be $e_{T}=500$ m. From this table, it can be seen that the filters with DBT measurements perform better than those with BOT. We also see that for known measurement noise covariance, the filters have lower track loss. It can be also observed that the orthogonal polynomial filters have almost similar track loss to the square root filters.

Finally, we compare the filtering performance in terms of their execution time. The relative execution times of all filters with respect to the EKF for both BOT and DBT are reported in Table 4. From this table, we see that the execution times of the GHO-EKF and SRGHF are almost three to four times those of the EKF. The CO-EKF and UO-EKF take nearly 50% more execution time than the EKF, whereas the computation time of the SRCKF and SRUKF is nearly twice that of the EKF. We also observe that for both BOT and DBT measurements, the orthogonal polynomial filters have lower computation times than their respective square root deterministic sample filters. As expected, the adaptation of measurement noise covariance further increases the execution time of the filters.

**Figure 14:**
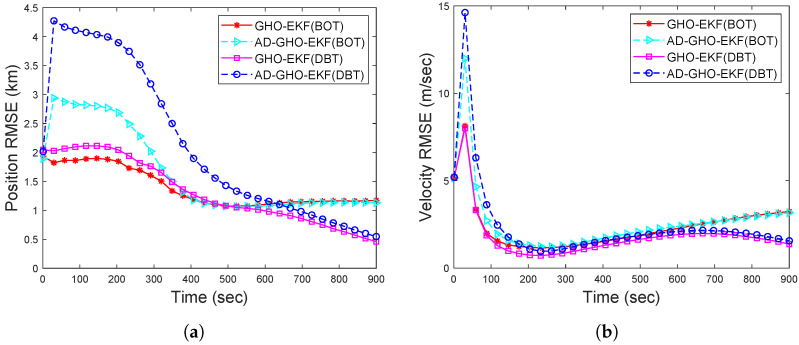
RMSEs of the (**a**) position; (**b**) velocity of the BOT and DBT for maneuvering target.

To assess how our proposed algorithms work in the presence of a target maneuver, we consider a target that is maneuvering with the constant turn rate (CT) model, as described in Section 2.1.2, with known ω=−0.015°/s, which is shown in Figure 1a. Initially, we assume that the estimators know the target maneuvering model and check the performance of all the filters. We see that all the filters perform with similar accuracy, as described in Figure 2, Figure 3, Figure 4, Figure 5, Figure 6, Figure 7, Figure 8, Figure 9, Figure 10, Figure 11, Figure 12 and Figure 13. Further, to study the robustness of these filters, we assume that the target is following a maneuver as described above, but the estimators are unaware of this dynamics and they use the constant velocity model to estimate the position and velocity of the target. To cope with such model mismatch, the process noise intensity of the filtering algorithm is increased to 100 $\tilde{q}$. The target and all the estimators are initialised as described in Section 4.3. Initially, we experiment with a 500 m track loss threshold, i.e., $e_{T}=500$ m, and it has been observed that the final track error in all the filters in this model mismatched case was above this threshold in most of the runs. Further, we calculate the percentage of track loss for all the filters considering $e_{T}=1500$ m and list them in Table 5. From this table, we note that DBT shows much less track loss than BOT and the adaptive filters in DBT show less than 1% track loss, while their non-adaptive counterparts exhibit zero track loss. However, for BOT, the percentage of track loss obtained from adaptive filters is almost double that of their non-adaptive counterparts. In Figure 14, we plot the RMSE of position and velocity obtained from the adaptive and ordinary GHO-EKF for both the bearings-only and Doppler-bearing tracking. Comparing Figure 5 with Figure 14, it has been observed that the position RMSE for the model mismatch case is converging to a slightly higher value than that of the the constant velocity scenario. Further, the RMSE obtained from DBT is lower compared to BOT, and the same obtained with GHO-EKF is lower than its adaptive counterpart.

## 5. Discussion and Conclusions

In this paper, we have proposed an adaptive orthogonal polynomial-based filter that can estimate the target trajectories along with the measurement noise error covariance. It is based on the linearisation of the measurement function using the first-order orthogonal polynomial approximation, and the coefficients of these polynomials are evaluated using the numerical integration technique. The proposed method is used to estimate a constant velocity target trajectory for two different tracking scenarios, each with bearings-only and Doppler-bearing measurements. The estimation accuracy of the filters is studied in terms of RMSE. We also use the percentage track loss, computation time, average NEES, and bias norm to compare the performance of the filters. The robustness of the proposed algorithm is studied when there is a mismatch between the target and the filter-assumed dynamics. From the simulation results, it can be deduced that the proposed filters consistently outperform the Taylor series-based EKF and provide comparable performance with their square root counterparts of the deterministic sample point filters with a reduced computational cost. Moreover, the proposed filters with CV model target dynamics can estimate the track of a lightly maneuvering target with acceptable accuracy. Target motion analysis with unknown sensor misalignment remains under the scope of future works.

## Figures and Tables

**Table 1:** Flops count of the implemented filters for BOT and DBT.

Filter	BOT		DBT	
	**Ordinary**	**Adaptive**	**Ordinary**	**Adaptive**
EKF	409	516	589	914
SRCKF	1197	1548	1475	2053
CO-EKF	775	966	1011	1440
SRUKF	1323	1711	1659	2282
UO-EKF	817	1008	1065	1444
SRGHF	8090	11,142	10,299	14,162
GHO-EKF	3913	4104	4953	5382

**Table 2:** Parameters used in tracking scenarios.

Parameters	Scenario I	Scenario II
Initial range	16.1 km	16.1 km
Target speed	35 knots	35 knots
Target course	−160°	−160°
Observer speed	18 knots	18 knots
Observer initial course	160°	180°
Observer maneuver	61th to 420th s	181th s
$\sigma_{r}$	2 km	2 km
$\sigma_{s}$	2 knots	2 knots
$\sigma_{\theta }$	1.5°	1.5°
$\sigma_{f}$	1 Hz	1 Hz
$\sigma_{c}$	$\pi /\sqrt{5}$	$\pi /\sqrt{5}$
$\tilde{q}$ (km${}^{2}$/s${}^{3}$)	$9\times 10^{-12}$	$9\times 10^{-12}$
*T*	1 s	1 s

**Table 3:** Percentage track loss of different filters of the BOT and DBT for both scenarios.

	Scenario I		Scenario II	
Filter	BOT	DBT	BOT	DBT
EKF	2.30	0	6.73	0
AD-EKF	9.86	2.27	17.35	2.09
SRCKF	2.25	0	5.72	0
CO-EKF	2.03	0	6.17	0
AD-SRCKF	8.28	1.3	16.51	0.91
AD-CO-EKF	8.78	1.35	16.23	1.01
SRUKF	2.16	0	6.12	0
UO-EKF	2.22	0	6.26	0
AD-SRUKF	8.48	1.41	17.73	1.05
AD-UO-EKF	8.69	1.38	15.61	0.95
SRGHF	1.86	0	6.35	0
GHO-EKF	2.24	0	6.27	0
AD-SRGHF	8.20	0.72	16.96	0.74
AD-GHO-EKF	8.35	0.80	16.41	0.80

**Table 4:** Relative execution times of the different filters for BOT and DBT.

Filter	BOT		DBT	
	**Ordinary**	**Adaptive**	**Ordinary**	**Adaptive**
EKF	1.00	2.14	1.28	2.40
SRCKF	1.84	3.72	2.14	4.02
CO-EKF	1.51	2.70	1.95	2.92
SRUKF	1.88	3.80	2.28	4.09
UO-EKF	1.53	2.78	2.03	2.99
SRGHF	6.53	10.35	10.52	14.13
GHO-EKF	4.16	5.97	5.64	7.75

**Table 5:** Percentage of track loss for BOT and DBT when model mismatch occurs.

Filter	BOT		DBT	
	**Ordinary**	**Adaptive**	**Ordinary**	**Adaptive**
EKF	13.34	29.72	0	0.35
SRCKF	12.30	26.95	0	0.25
CO-EKF	12.40	26.94	0	0.26
SRUKF	12.62	26.80	0	0.28
UO-EKF	12.04	27.08	0	0.25
SRGHF	11.55	26.57	0	0.20
GHO-EKF	11.53	26.62	0	0.20

## Data Availability

Not applicable as all the results are generated with simulated data.
